# Dramatic response to dabrafenib and trametinib combination in a BRAF V600E-mutated cholangiocarcinoma: implementation of a molecular tumour board and next-generation sequencing for personalized medicine

**DOI:** 10.3332/ecancer.2014.479

**Published:** 2014-11-06

**Authors:** Arturo Loaiza-Bonilla, Erica Clayton, Emma Furth, Mark O’Hara, Jennifer Morrissette

**Affiliations:** Abramson Cancer Center of the University of Pennsylvania, Perelman Center for Advanced Medicine, Civic Center Boulevard, Philadelphia, PA 19104, USA

**Keywords:** BRAF, cholangiocarcinoma, dabrafenib, exceptional responder, genomic medicine, MEK, molecular tumour board, next-generation sequencing, personalized medicine, trametinib

## Abstract

This is the case of a 47-year-old woman diagnosed with chemotherapy and radiation-refractory BRAF V600E mutant, poorly differentiated intrahepatic cholangiocarcinoma (ICC), with multiple metastatic lesions within the liver, lungs, pleura, and bone, stage IV. Discussion of her malignancy’s next-generation sequencing genomic information at a multidisciplinary molecular tumour board took place. The patient was considered a suitable candidate for dual BRAF and MEK inhibition, with the intent to prolong her survival and optimize the quality of life. We report her excellent tolerance and exceptional response to dual therapy with dabrafenib and trametinib, including symptomatic and sustained near-complete radiological improvement. We also briefly review the current knowledge of the genomics of cholangiocarcinoma with a focus on BRAF mutations, and make a point of the importance of the establishment of a molecular tumour board for personalized genomic medicine approaches. To our knowledge, this is the first reported case of the use of personalized genomic information for the successful management of a patient with ICC, and it is also the first description of dual BRAF and MEK targeted therapy in this malignancy, leading to what is considered an exceptional response.

## Background and case report

A 47-year-old woman with a history of smoking and transient gestational diabetes was in her usual state of health until July 2013 when she started to complain of occasional, self-limited abdominal pain. She consulted her primary care physician who recommended a computed tomography (CT) scan of the abdomen and pelvis to rule out diverticulitis. Performed in August 2013, it incidentally revealed several hypodense lesions in both lobes of the liver, the largest located in the left hepatic lobe and measuring about 7 × 6.4 × 4.6 cm. Due to these findings, she underwent upper esophagogastroduodenoscopy (EGD) and colonoscopy, both of which were unremarkable. A screening mammogram and vaginal ultrasound were also reported as normal. Further workup included a magnetic resonance imaging (MRI) scan of the abdomen with gadavist in August 2013, which confirmed the CT-scan findings. Given non-homogeneous enhancement, the differential suggested included atypical haemangiomas, adenomas, and malignancy. A [18F] fluorodeoxyglucose (FDG) positron emission tomography (PET)/CT scan reported multiple FDG-avid hypermetabolic lesions within the liver, the largest measuring 8 cm in diameter. The patient was subsequently referred to the Abramson Cancer Center (ACC) GI oncology clinic for evaluation and treatment recommendations.

**Figure 1.**
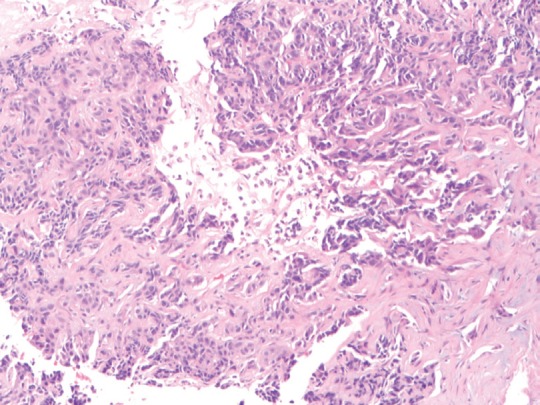
(10× magnification) biopsy of the liver lesion shows a poorly differentiated neoplasm in a desmoplastic stroma.

**Figure 2.**
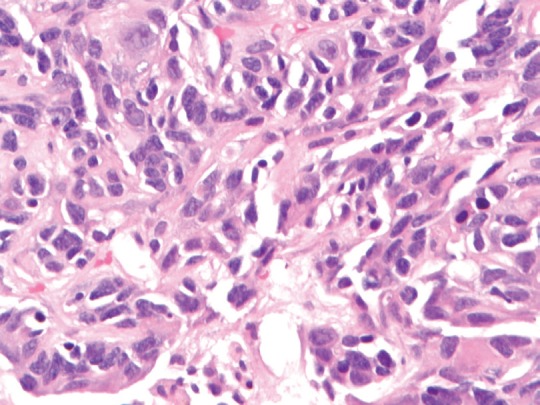
(40× magnification). High-power magnification of the tumour shows pleomorphic cells without significant mitotic activity, inclusions, or pigment.

**Figure 3.**
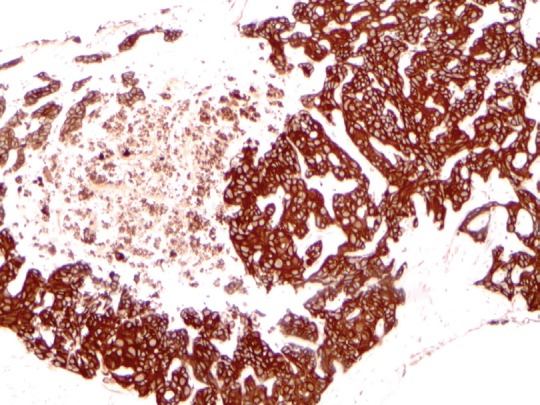
Immunohistochemical workup shows the tumour was strongly and diffusely positive for CK7 (10× magnification).

A review and discussion of her case was held by the multidisciplinary tumour board at ACC. A CT-guided biopsy of the left liver lobe mass was performed in mid-September 2013. Pathology described a poorly differentiated adenocarcinoma in the liver. The biopsy showed highly atypical malignant cells infiltrating liver parenchyma with a solid growth pattern with vague glandular formation, areas of necrosis, and a desmoplastic stromal response. The tumour cells were pleomorphic in size with polygonal to focally spindled morphology and large nuclei with variably prominent nucleoli and chromatin pattern, and ample eosinophilic cytoplasm. Mitoses were infrequent, and there were no apparent intracytoplasmic or intranuclear inclusions, single cell necrosis, or pigment. Immunohistochemical stains were performed and showed that the tumour was strongly and diffusely positive for CK7 with focal weak positivity for CEA. The tumour was negative for CK20, CA-125, TTF-1, NapsinA, Brst2, GATA3, oestrogen receptor (ER), progesterone receptor (PR), and Her2 (Figures 1–3). Serum tumour markers showed elevated chromogranin A, carcinoembryonic antigen, and CA 15-3. A final diagnosis of poorly differentiated intrahepatic cholangiocarcinoma (ICC) was made.

**Figure 4.**
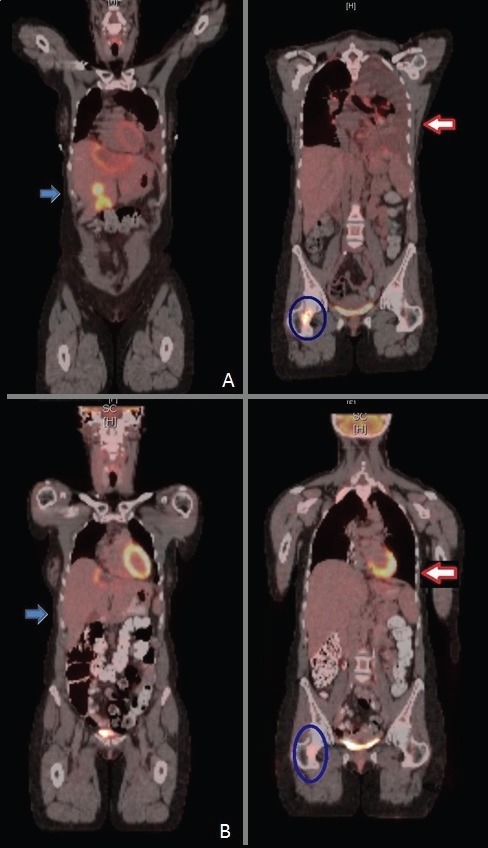
PET scan before (upper panel A) and two months after dabrafenib and trametinib combination (lower panel B), showing improvement in liver metastasis (blue arrow), resolution of malignant left pleural effusion and lung nodules (red arrow) and improvement of bone metastasis (blue circle).

Given the diagnosis of non-resectable ICC with oligometastatic liver disease the patient was considered to undergo palliative liver-ablative radiation therapy with concomitant chemotherapy (infusional 5-Fluorouracil 200 mg/m2/day seven days per week) as part of a potential multimodal approach. The patient completed this course of therapy in late December 2013, at which time she was planned to start systemic chemotherapy (a platinum/gemcitabine combination). In January 2014, the patient presented to an outside hospital due to new onset shortness of breath, and a CT of the chest revealed a large left pleural effusion as well as left lower lobe and distal pulmonary artery filling defects consistent with a subacute/chronic pulmonary embolism. She was started on Enoxaparin systemic anticoagulation. She also underwent a left-sided thoracentesis and cytology revealed metastatic adenocarcinoma. The MRI of the abdomen reported multiple hepatic masses (largest 8.4 × 7.3 × 5.5 cm in size). The PET/CT scan in February 2014 revealed at least four metabolically active hepatic metastases, increased in size when compared to the previous study. There was also evidence of new hypermetabolically active mediastinal and bilateral hilar lymph nodes, a 1.7 cm right lower lobe consolidation/nodule, a 3.1 cm left lower lobe mass, new active osseous metastases in the right first rib and right femoral neck, and other scattered metabolic areas of consolidation in the right lung (Figure 4, panel A).

Next-generation sequencing testing of this patient’s tumour was ordered for genomic analysis in an attempt to determine potential therapeutic targets. This test was developed and its performance characteristics determined by the University of Pennsylvania’s Center for Personalized Diagnostics Laboratory as required by the Clinical Laboratory Improvement Amendments (CLIA) 1988 regulations. Pursuant to the requirements of CLIA 1988, this laboratory has established and verified the test’s accuracy and precision.

## Methodology

Genomic DNA was extracted from the submitted specimen (paraffin-embedded tissue block from initial diagnostic liver biopsy) according to manufacturer’s instructions (Qiagen, Inc.). Targeted analysis for mutations in the regions specified in this testing panel was achieved by the enrichment of those genomic loci using the Illumina Truseq Amplicon Assay. Sequencing of enriched libraries was performed on the Illumina MiSeq platform using multiplexed, paired-end reads with Version 2 chemistry. Analysis and interpretation utilized a customized bioinformatics process. All variants listed are with reference to the hg19 Genome build. Variants are reported according to Human Genome Variation Society (HGVS) nomenclature and classified into three categories: pathogenic, variants of uncertain significance, and benign. Categorization of variants was dependent upon literature review, variant existence in a variety of publically available databases, including the Single Nucleotide Polymorphism Database (dbSNP), the Catalogue of Somatic Mutations in Cancer (COSMIC), and the 1,000 genome project. Variants classified as benign are not listed in the final report.

This assay detects single nucleotide variants and small insertions or deletions (*indels*). Large or complex *indels*, inversions, translocations, gene amplifications, copy number changes or other complex genomic mutations may not be detected by this assay. Variants existing outside the target regions cannot be detected. Only variants in the exonic regions of the gene panel are reported. This assay does not determine variant causality, or whether a variant is inherited or somatically acquired. This assay will detect variants representing at least 10% of the total sequence reads at a given genomic position. During test validation, the choice of parameters was optimized to maximize sensitivity and specificity.

Solid tumour-sequencing panel included sequence analysis of 47 genes (ABL1, AKT1, ALK, APC, ATM, BRAF, CDH1, CSF1R, CTNNB1, EGFR, ERBB2, ERBB4, FBXW7, FGFR1, FGFR2, FGFR3, FLT3, GNA11, GNAQ, GNAS, HNF1A, HRAS, IDH1, JAK2, JAK3, KDR, KIT, KRAS, MET, MLH1, MPL, NOTCH1, NPM1, NRAS, PDGFRA, PIK3CA, PTEN, PTPN11, RB1, RET, SMAD4, SMARCB1, SMO, SRC, STK11, TP53, and VHL). Genomic regions not meeting criteria for variant calling include the genomic loci for which the depth of coverage did not meet our minimum criteria of 250 reads at all positions within those loci. Since the sensitivity and specificity of our assay were determined by achieving a minimum depth of coverage of 250 reads, regions not meeting this criterion cannot be guaranteed to be mutation negative. The depth of coverage is dependent upon the starting amount of DNA for a given region that could be affected by underlying chromosomal copy number changes. Since the genomic loci targeted in this assay are enriched using loci-specific primers, patient variants existing within those primer sequences may also cause a region to fail enrichment and therefore not achieve 250x coverage. The design of the panel was based on the literature at the time of development, either the full length of a gene or the mutational hot spots of a gene are targeted.

## Results

This analysis reported a mutation in BRAF p.V600E c.1799T > A (8819 reads out of a total 16,712 sequence reads for an allele frequency of 52.77). After multidisciplinary discussion at our molecular tumour board, it was determined that the high allele frequency may deem the malignancy potentially vulnerable to BRAF inhibition, ideally in combination with a MEK inhibitor. The patient was started on palliative, off-label dabrafenib 50 mg twice daily and trametinib 2 mg once daily, to continue until disease progression or unacceptable toxicity. Cycle 1 was administered early February 2014. Denosumab and vitamin D with calcium supplementation were added to prevent skeletal-related events, and she received one dose of external beam, proton-based radiation therapy (8 Gy) to her right femoral lesion. Restaging CT scans in March 2014 (less than 4 weeks after starting treatment) revealed spontaneous resolution of the left pleural effusion and almost complete resolution of the mass-like nodules and hilar adenopathy in the right lung. The liver lesions also improved (the dome lesion decreased from 1.9 cm to 1.1 cm, the large left lobe lesion from 9.1 cm × 6.8 cm to 7.6 cm × 5.7 cm, and the lesion in segment V from 1.8 cm to 1.4 cm). The bone lesions appeared sclerotic due to treatment response.

Repeat restaging CT scans and PET/CT scan in mid-April 2014 reported continuous response to treatment with a residual reactive lesion in the left lobe of the liver adjacent to the left dominant mass (which was no longer FDG-avid), and the resolution of activity of other previous liver metastases, lung lesions and skeletal metastases (Figure 4, panel B). After 8.5 months (34 wks) of therapy, she remains almost completely asymptomatic, with the exception of some episodes of chills and shaking at night and one episode of self-limited rash over her left ankle that was diagnosed as erythema nodosum. All of these symptoms have been controlled with ibuprofen twice daily as needed. Initially, she had also developed grade 1 dizziness and blurry vision when reading small print, but eventually resolved. Her cough resolved along with her hip pain. Liver-targeted ablative techniques (transarterial chemoembolization or radiofrequency ablation) are now planned in order to treat the remainder of her oligometastatic disease within the liver. She continues to follow with dermatology and ophthalmology monthly as planned.

## Discussion

Liver cancer is the third leading cause of cancer mortality worldwide, and biliary tract cancers account for approximately 10–20% of hepatobiliary neoplasms, with about 9,000 cases diagnosed in the US each year. Cholangiocarcinoma is the second most common type after hepatocellular carcinoma (HCC), accounting for 10–15% of all primary liver malignancies. [1, 2]

Cholangiocarcinoma is clinically sub-classified as intrahepatic (ICC) when it arises from the small bile ducts within the liver. When arising from the ductal epithelium of the confluence of the main left and right hepatic ducts, or distal in the bile ducts it is considered extrahepatic (ECC). This classification has allowed for identification of substantial distinct genetic and histologic features, risk factors, epidemiology, presentation, and outcomes [3–5]. Surgical resection represents the only curative modality for these patients; however, most cases are diagnosed at advanced stages, with median overall survival of less than 2 years from the time of diagnosis despite palliative chemotherapy [5–10].

In an effort to improve the outcomes of these patients, several molecular studies of ICC have been performed, aiming to detect targetable oncogenic-driving mutations, as well as predictive and prognostic genomic markers of response [11–18].

Ross *et al* compiled the largest cohort of ICC cases to date, where DNA sequencing of hybridization-captured libraries was performed for 3,320 exons of 182 cancer-related genes, and 36 introns of 14 genes frequently rearranged in cancer. Two thirds of patients in this study harboured targetable genomic alterations that have the potential to personalize therapy selection for individual patients [13].

The most commonly described activating mutations in ICC genomic studies are KRAS [14, 15], HER2 [16], MET [17], as well as hotspot activating missense mutations in genes downstream to epidermal growth factor receptor (EGFR), such as the v-raf murine sarcoma viral oncogene homolog B1 gene (BRAF) [18].

BRAF is located on chromosome 7q34. It is a member of the Raf kinase family that plays a critical role in regulating the cell proliferation through the mitogen-activated protein (MAP) kinase pathway. Mutant BRAF has been implicated in the pathogenesis of many cancers; however, it can also be seen in benign conditions. A majority of mutations in BRAF occur at codon 600 with the most common changes, including V600E, V600K, and V600R. Other missense substitutions have also been reported, including rare mutations, such as K601E, L597R, and L597S [19, 20].

BRAF mutation-driven ICCs have been reported at a frequency between 0% and 22% [21–25]. The highest frequency of such mutations was reported by Tannapfel *et al*, who identified activating BRAF missense mutations in 15 out of 69 ICC (22%) [18]. Sia *et al* performed whole-genome expression profiling in a final cohort of 149 ICC patients. Mutations at exon 15 of BRAF were found in five patients (5 of 141; 4%) [21]. Andersen *et al* profiled the transcriptomes from 104 surgically resected cholangiocarcinomas. The mutational status of KRAS/ BRAF was significantly associated with poor prognosis, and the BRAF V600E mutation was present in 1/69 cases (1.5%) [22]. Conversely, another study by Xu *et al* did not find any BRAF mutations in a series of 34 ICCs, a finding also reported by others. This highlights the genomic heterogeneity of such tumours [23–25]. Another study using genome-wide analysis and clinical correlation of chromosomal and transcriptional mutations in 34 biliary tract cancer specimens reported 2,354 genes with altered expression in ICC, revealing the exceptional diversity of mutational findings between individual patient specimens [12, 26].

In addition, Sia *et al* identified 2 main biological classes of ICC. A group named the “proliferation class” (62% of ICCs in this study) had specific copy-number alterations. These were high-level amplifications in 5 regions, including 1p13 (9%) and 11q13.2 (4%), and several focal deletions, such as 9p21.3 (18%) and 14q22.1 (12% in coding regions for the SAV1 tumour suppressor). Many features of the poor-prognosis signatures for HCC were also noted in this class, as well as a worse outcome (*P* < 0.001) when compared to the other group of ICCs. It was also characterized by the activation of oncogenic signalling pathways (including RAS, MAP kinase, and MET), DNA amplifications at 11q13.2, deletions at 14q22.1, mutations in KRAS and BRAF [21].

The second group described by Sia was the ‘inflammation class’ (38%), which was characterized by the activation of inflammatory signalling pathways, overexpression of cytokines, and STAT3 activation [21]. Given these findings and evidence of significant pleiotropic and biologic heterogeneity, there is a clear need for appropriate classification of subsets of ICC patients during treatment choice selection, as these signatures seem not only prognostic but also predictive for response to targeted therapy.

## Rationale for dual BRAF targeting

Since the advent of the concepts of ‘oncogene-addiction phenomenon’ and compensatory or escape pathways, the development of personalized-targeted medicine leading to synthetic lethality has become a subject of active investigation [27–29]. The substantial genomic variability noted in ICC provides the rationale for such approaches based on each patient’s ICC’s particular genetic makeup [21, 26].

The MAP kinase (MAPK) pathway comprises several important targets for targeted therapy. Specific mutations in BRAF can stimulate this pathway, and the presence of a V600E or K mutation predicts responsiveness to BRAF inhibitors or MEK inhibition. Three Food and Drug Administration (FDA)-approved agents have demonstrated significant clinical benefits and been approved for use in patients with metastatic melanoma harbouring BRAF mutations: the BRAF inhibitors dabrafenib and vemurafenib and the potent, highly specific inhibitor of MEK1/ MEK2 trametinib. Inhibition of the MAP kinase pathway in patients whose tumours contain a V600 mutation in the BRAF gene using either a BRAF inhibitor or a MEK inhibitor is an important target in personalized medicine, particularly in tumours that do not respond to a systemic chemotherapy regimen, or when a standard treatment is not well defined [30–32].

When used as single agents, the BRAF inhibitor dabrafenib and the MEK inhibitor trametinib demonstrated superior progression-free survival (PFS) versus chemotherapy in patients with BRAF V600E/K mutant metastatic melanoma. There seems to be an advantage in PFS and toxicity profile when a BRAF inhibitor and a MEK inhibitor are used in combinations, and studies indicate that there is a reduced incidence of skin toxicity, including the development of cutaneous squamous carcinoma associated with BRAF inhibition. This presumably results from MEK inhibition that blocks the escape pathway and the subsequent paradoxical activation of the MAPK pathway. A phase I/II study [31] demonstrated the feasibility of combining full doses of dabrafenib (150 mg twice daily) and trametinib (2 mg daily); this resulted in improvement in overall response rate (ORR) and PFS, as well as reducing dermatologic toxicity, manifested by squamous cell carcinoma (including keratoacanthoma).

In the phase III component of that trial, 423 patients with unresectable or metastatic BRAF V600E/K mutant melanoma were randomly assigned to dabrafenib (150 mg twice daily) plus trametinib (2 mg once daily; *n* = 211) or dabrafenib plus placebo (*n* = 212) [33]. The primary endpoint was investigator-assessed PFS. Secondary endpoints were overall survival (OS), ORR, duration of response, and safety. Cross-over was not allowed. The study had 95% power and a one-sided *α* = 0.025 to detect a PFS hazard ratio (HR) of 0.59. After a median follow-up of 9 months (range, 0–16 months), the HR for 6-month investigator-assessed PFS was 0.75 (95% CI: 0.57–0.99; *P* = 0.035), in favour of dabrafenib plus trametinib. Median PFS was 9.3 months for the dabrafenib plus trametinib arm versus 8.8 months for the dabrafenib plus placebo arm. Confirmed ORR was 67%, with a complete response of 10% for the dabrafenib plus trametinib arm, versus 51% with a complete response of 9% for dabrafenib plus placebo (*P* = 0.0015). The interim OS HR was 0.63 (95% CI: 0.42–0.94; *P* = 0.023), in favour of dabrafenib plus trametinib. The rate of adverse events (AEs) was similar for both arms; however, more patients in the dabrafenib plus trametinib arm had increased incidence (51% versus 28%) and severity (grade 3, 6% versus 2%) of pyrexia compared with dabrafenib plus placebo. The combination also resulted in less cutaneous squamous carcinoma (2% versus 9%) and hyperkeratosis (3% versus 32%), respectively, compared with dabrafenib and placebo; both are considered BRAF-inhibitor related AEs [33].

Based on the aforementioned, for patients suffering from metastatic BRAF V600E/K mutant malignancies who are ineligible for clinical trials and who are candidates for targeted therapy, it seems biologically advantageous to start with the combination of dabrafenib and trametinib rather than a single agent.

## Establishing a molecular tumour board

Along the exponential growth in our knowledge of critical genomic alterations in cancer, there is an accelerated need to incorporate such data into the development of personalized regimens tailored to the specific genetic makeup of individual patients and their malignancies. The fast pace and affordability of DNA sequencing and other genomic techniques are leading this new era of personalized medicine, with many patients presenting to our oncology practices with several voluminous reports containing genomic data provided by different companies. The heterogeneity and variability of such reports call for the development of a multidisciplinary and more standardized method to analyse these data, in order to translate it into effective therapies for our patients.

Following this national trend, the Abramson Cancer Center at the University of Pennsylvania developed a multidisciplinary molecular tumour board in 2013, meeting twice a month. These efforts have already been reported as successful in other centres, leading to a better understanding of how to establish an institutional personalized cancer programme. For example, the University of California San Diego Moores Cancer Center’s molecular tumour board was able to identify 12 chemotherapy-refractory patients who were candidates for a personalized-targeted therapy based on their genomic information, with three patients showing a partial response [34].

These experiences of ‘extraordinary responders’ provide the foundation of basket trial designs involving targeted therapies. Unlike several other solid tumours, no molecular-targeted agents have been approved for the treatment of ICC and many other malignancies. By incorporating the genotype-to-phenotype concept, it is possible to enroll our patients harbouring specific mutations in pertinent phase II and III trials exploring molecular-targeted therapies or chemotherapeutical regimens, rather than a disease-specific (or phenotype-to-genotype) approach. The National Cancer Institute and other institutions are paving the way for those efforts, including the Exceptional Responders Initiative, the Molecular Analysis for Therapy Choice (NCI MATCH) among others [35, 36].

## Conclusion

To our knowledge, this is the first reported case of the use of personalized genomic information for the successful management of a patient with ICC, and it is also the first description of dual BRAF-targeted therapy in this malignancy, leading to what is considered an exceptional response.

While we continue advancing in our understanding of genomic personalized approaches and technologies, it is clear that new ways to treat patients will continue to emerge, accompanied by an equal number of challenges and questions. The applicability and final implementation of these efforts will certainly require extensive multi-institutional collaborations. Personalized genomic medicine holds the promise of leading this unparalleled momentum in translational oncology, and will certainly positively change the current treatment paradigm.

## Conflicts of interest

The authors declare that they have no conflict of interest.

## Authors’ contributions

All authors contributed to this manuscript and reviewed its final version.
